# Relationship between sleep-related impairment and cognitive functioning among breast cancer survivors

**DOI:** 10.1007/s11764-026-02057-7

**Published:** 2026-06-13

**Authors:** Victoria M. Telles, Victoria Mac, Rowena Tam, Marisa Torres-Ruiz, Sheri J. Hartman

**Affiliations:** 1 Herbert Wertheim School of Public Health and Human Longevity Science, University of California, San Diego, La Jolla, CA USA; 2 Moores Cancer Center, University of California, San Diego, La Jolla, CA USA; 3 San Diego State University, San Diego, CA USA; 4 Doctor of Physical Therapy Program, San Diego State University, San Diego, CA USA

**Keywords:** Breast cancer survivorship, Cancer, Cognitive function, Sleep-related impairment, Cancer-related cognitive decline

## Abstract

**Purpose:**

Sleep-related impairment and cancer-related cognitive decline (CRCD) are common, disruptive symptoms affecting breast cancer survivors. This study examined the association between sleep-related impairment and both self-reported and objective cognitive functioning in breast cancer survivors following treatment.

**Methods:**

Participants (*N* = 253) from the *I Can! Improving Cognition After Cancer* study completed the NIH Toolbox Digit Symbol test to measure Processing Speed and a battery of neurocognitive tests of Memory, Executive Functioning, and Attention domains. Self-reported cognition was assessed with the PROMIS Cognitive Function and Abilities scales. Sleep-related impairment was measured by the PROMIS Sleep-Related Impairment scale. Multiple linear regression models were used to examine the associations between sleep-related impairment and the six cognitive outcomes, adjusting for demographic and cancer treatment factors.

**Results:**

Participants were on average 59 (SD = 8.9) years old, majority identified as White (77%) and were college graduates (42%). Fifty-seven percent of participants underwent chemotherapy, 80% received hormone therapy, and 81% were treated with radiation. Greater sleep-related impairment was significantly associated with worse executive function (*β* = −0.01, 95% CI: −0.022, −0.000, *p* = 0.04), worse cognitive abilities (*β* = −0.28, 95% CI: −0.375, −0.184, *p* < 0.001), and worse cognitive function (*β* = −0.25, 95% CI: −0.323, −0.167, *p* < 0.001). No significant associations were observed for memory, attention, or processing Speed.

**Conclusion:**

Greater sleep-related impairment was associated with poorer self-reported cognitive functioning and modest differences in executive functioning among breast cancer survivors, underscoring the importance of addressing sleep-related impairment in this population.

**Implications for Cancer Survivors:**

Assessing sleep-related impairment during survivorship care may help identify survivors experiencing cognitive difficulties and related daytime functional concerns.

**Supplementary Information:**

The online version contains supplementary material available at https://doi.org/10.1007/s11764-026-02057-7.

## Introduction

Cancer-related cognitive decline (CRCD) is a pervasive complication reported by up to 75% of breast cancer survivors (BCS) following treatment [1, 2]. CRCD impacts several cognitive domains including problems with memory, executive functioning, verbal fluency, processing speed, and attention [2–5]. These complications can disrupt an individual’s ability to work, daily functioning, social relationships, and overall quality of life [2, 6–8]. However, emerging research suggests that sleep-related difficulties may represent a robust and modifiable risk factor associated with cognitive impairment in BCS [9–13].

Poor sleep has been associated with worse *perceived* cognitive functioning among cancer survivors, though its link to *objective* cognitive impairment remains inconsistent [13, 14]. Sleep-related difficulties, including sleep disturbances, insufficient sleep duration, and poor sleep quality, contribute to various conditions, such as insomnia, sleep apnea, excessive daytime sleepiness, and sleep-wake disorders [15, 16]. Although a recent systematic review found that sleep-related symptoms were consistently linked to worse neurocognitive functioning, its relationship to objective cognitive impairment was less evident [14]. Some studies have shown that sleep-related difficulties are most strongly associated with deficits in objective verbal functioning, memory, and executive function; however, evidence is limited [10, 14, 17]. Given these mixed findings, further research is needed to further elucidate the relationship between sleep-related impairment and objective cognitive performance in this population.

Despite the conflicting evidence between objective and perceived cognitive impairment outcomes, both are important to capture, as each provides relevant information for BCS functioning [18–20]. Objective cognitive impairment measures are subject to practice effects (i.e., benefits from familiarity with test format) [21] and may not capture the real-world cognitive challenges experienced by patients [22]. Conversely, self-reported cognitive complaints can often be bidirectionally associated with psychological distress (i.e., cognitive complaints are associated with distress and vice versa) [6, 22]. Thus, there is a need to understand the multifactorial nature of perceived and objective cognitive impairment, including the potential role of sleep-related symptoms and daytime impairment among BCS.

While a substantial body of research in the general population suggests that sleep-related symptoms and disturbances are associated with cognitive dysfunction [10, 23–26], research evaluating this effect on BCS post-treatment is limited [9, 10, 23]. Sleep-related impairment and associated daytime dysfunction may exacerbate the neurocognitive effects resulting from chemotherapy and endocrine therapy, making BCS particularly vulnerable to cognitive difficulties. Thus, a robust understanding of the association between sleep-related impairment and both objective and perceived cognitive impairment among BCS post-treatment is essential to inform clinical and survivorship care. Additionally, despite the persistent sleep-related difficulties reported by cancer survivors [15, 16, 27, 28], it is frequently overlooked as a modifiable lifestyle factor that can reduce the risk of other symptoms [29], including cognitive functioning, fatigue, depression, anxiety, pain, and immune function during survivorship care. Our primary aim for this study was to examine the relationship of sleep-related impairment with objective (memory, executive functioning, attention, and processing speed) and self-reported (cognitive function and abilities) cognitive outcomes. We hypothesized that greater sleep-related impairment would be associated with worse performance on objective neuropsychological tests and worse perceived cognition.

## Methods

### Participants

We analyzed baseline data from breast cancer survivors who participated in a 12-month randomized controlled trial titled “I Can! Improving Cognition After Cancer.” A comprehensive description of the study’s design and rationale has been detailed in prior publications [3]. The I Can! study enrolled female breast cancer survivors who had been diagnosed with stage 1 to 3 breast cancer within 5 years of their enrollment date. Eligible participants were at least 40 years old, had completed all active cancer treatments (e.g., chemotherapy, radiotherapy) at least 6 months prior to enrollment, and had a history of endocrine therapy and/or chemotherapy. Additional criteria included engaging in fewer than 60 min of moderate to vigorous physical activity per week (self-reported) and having subjective cognitive concerns, defined as a score of 4 or higher on a 0–10 cognition scale [30]. Participants were also required to have access to a Fitbit-compatible device with internet connectivity. Survivors with medical conditions that might present safety risks during unsupervised physical activity, those on tamoxifen or an aromatase inhibitor and planning to discontinue within 6 months, and individuals unable to commit to the 12-month duration of the study were excluded from the study. Baseline data collection occurred between September 2019 and December 2022, with analyses conducted from January to October 2024. The study protocol received approval from the UC San Diego Office of IRB Administration, and all participants provided written informed consent.

The majority of participant recruitment was conducted through cancer registry lists from the California Cancer Registry and the UC San Diego Healthcare electronic medical record system, with an IRB-approved HIPAA waiver facilitating medical record screening. Research activities were conducted in the UC San Diego Moore’s Cancer Center, the primary outpatient cancer care facility within the UC San Diego Healthcare System. Participants received a $20 incentive upon successful completion of all baseline measures.

### Measures

#### Assessment of sleep-related impairment 

Sleep-related impairment was evaluated using the Sleep-Related Impairment scale from the Patient-Reported Outcomes Measurement Information System (PROMIS) battery. Participants engaged in computerized adaptive testing (CAT), where responses to initial items informed the selection of subsequent questions [31]. Results were reported as normative T-scores. Higher scores reflect greater impairment in sleep-related functioning.

### Objective cognitive outcomes (memory, executive functioning, attention, processing speed)

Cognitive outcomes of memory, executive functioning, attention, and processing speed were assessed through a mixture of objective cognitive tasks and were combined into z-scores. The memory domain was evaluated using the NIH Toolbox Picture Sequence Memory and List Sorting Working Memory Tests [32], along with two scores from the Hopkins Verbal Learning Test-Revised (HLVT-R): summary of the three immediate recall scores and the delayed recall score [33]. Executive functioning was measured by completion times on the Trail Making Tests—Trails B [34] and the NIH Toolbox Dimensional Change Card Sort Test [32]. The attention domain was assessed with the NIH Toolbox Flanker Inhibitory Control and Attention Test [32] and four subscales from the Continuous Performance Test (CPT-3): Detectability (*d*’), Variability, Hit Reaction Time Block Change, and Hit Reaction Time InterStimulus Intervals Change [35]. Processing speed was measured using the Oral Symbol Digit test from the NIH Toolbox Cognition [36, 37].

### Self-reported cognitive outcomes (cognitive function and cognitive abilities)

Cognitive function and abilities were gauged through self-reported surveys utilizing the computer adaptive testing form of the PROMIS Cognitive Function and Cognitive Abilities scale. These scales assess perceptions of cognitive functioning across various domains, including mental acuity, concentration, verbal and nonverbal memory, and verbal fluency [38]. The Cognitive Function scale assesses negative aspects of cognition, such as difficulties or impairments (e.g., “I have trouble forming thoughts” and “My thinking has been slow”). The PROMIS Cognitive Abilities scale captures positive self-assessments of the presence and quality of cognitive abilities, such as “I have been able to think clearly” and “My mind has been as sharp as usual.” Higher scores in these scales represent more of the concept being measured. The correlation between cognitive function and abilities in this sample was *r* = 0.64 (*p* < 0.001). Although highly correlated, prior research suggests that variation in factor loadings, item response patterns, and participant thinking processes indicate that the constructs tap distinct aspects of cognitive function and should be reported separately [38].

### Collection of breast cancer treatment, diagnosis, and demographic data

Data on breast cancer diagnosis and treatment (hormone therapy, chemotherapy, radiation) were extracted from medical records, while demographic information—including age, race, and ethnicity—was obtained through self-reported surveys.

#### Statistical analysis

Descriptive statistics were calculated for participant demographics and breast cancer treatment characteristics. Continuous variables were reported as means and standard deviations (SD), while categorical variables were presented as counts and percentages (%). T-scores and SDs were calculated for self-reported cognition and sleep-related impairment. Descriptive severity categories were generated for PROMIS Sleep-related Impairment and PROMIS Cognitive Function T-scores using published cut points for PROMIS adult measures [39]. For sleep-related impairment, T-scores < 55 were classified as within normal limits, 55–55.9 mild impairment, 60–69.9 moderate impairment, and ≥ 70 severe impairment. For cognitive function, T-scores > 45 were classified as within normal limits, 40–45 mild impairment, 30–39.9 moderate impairment, and < 30 severe impairment.

To calculate overall z-scores for each cognitive domain (memory, executive function, attention), individual test scores within each domain were first converted to z-scores using the sample’s overall baseline means and standard deviations. These z-scores were then averaged to derive a composite score for each domain. For components that were not already age-adjusted, residuals were obtained by regressing the cognitive outcome on age, and these residuals were standardized into z-scores. To ensure that higher composite scores indicated better cognitive function, z-scores for tests where lower scores indicated better performance were multiplied by −1 prior to averaging. Processing speed was analyzed as a single test outcome.

Multiple linear regression models were used to examine the associations between sleep-related impairment and the four objective cognitive outcomes (memory, executive function, attention, processing speed) as well as the two self-reported cognitive outcomes (cognitive abilities and cognitive function), adjusting for demographic covariates (age, race/ethnicity, education, marital status, income) and cancer treatment factors (chemotherapy, hormone therapy, radiation, years since diagnosis). Sensitivity analyses were also conducted only adjusting for age and education. All statistical analyses were conducted using R version 4.3.1 [40].

## Results

Table 1 presents the participant demographic and cancer treatment characteristics. On average, participants were 59 years old (SD = 8.9). The majority identified as White (77%) and non-Hispanic Latina (84%). Most were college graduates (42%), reported an annual household income above $100,000 (43%), and worked full-time (45%). Regarding cancer treatment characteristics, most participants underwent lumpectomy (62%), and 81% were treated with radiation. Additionally, 44% were treated with both chemotherapy and hormone therapy, 43% received hormone therapy but not chemotherapy, and 13% received chemotherapy but not hormone therapy. On average, participants were 3 years post-diagnosis (SD = 1.3) with nearly equal distribution between stage 1 (42%) and stage 2 (43%) diagnoses.

**Table 1:** Demographic and cancer treatment characteristics

Characteristic	*N* = 253
**Age, years, *n* (SD)**	59 (8.9)
**Ethnicity, *n* (%)**	
Non-Hispanic/Latina	212 (84%)
Hispanic/Latina	41 (16%)
**Race, *n* (%)**	
White	194 (77%)
Asian	27 (11%)
Black or African American	8 (3.2%)
American Indian or Alaska Native	3 (1.2%)
Native Hawaiian or Other Pacific Islander	1 (0.4%)
Other/multiple	20 (7.9%)
**Marital status, *n* (%)**	
Never married	12 (4.7%)
Divorced/separated/widowed	69 (27%)
Presently married or living with a partner	172 (68%)
**Income, *n* (%)**	
Less than $60,000	50 (20%)
$60,000 to $100,000	60 (24%)
Greater than $100,000	108 (43%)
Prefer not to answer	35 (14%)
**Education, *n* (%)**	
Some college or less	80 (32%)
College graduate	107 (42%)
Graduate degree (Master’s, Ph.D., M.D., J.D., etc.)	66 (26%)
**Employment status, *n* (%)**	
Not working for pay or wages	45 (18%)
Retired	58 (23%)
Working full-time (> 35 h per week)	114 (45%)
Working part-time (< 35 h per week)	34 (13%)
NA	2 (0.8%)
**Surgery type, *n* (%)**	
Lumpectomy	156 (62%)
Mastectomy	97 (38%)
**Radiation, *n* (%)**	206 (81%)
**Combined chemotherapy and hormone therapy, *n* (%)**	
Chemotherapy No, Hormone therapy Yes	108 (43%)
Chemotherapy Yes, Hormone therapy Yes	112 (44%)
Chemotherapy Yes, Hormone therapy No	33 (13%)
**Pathologic stage, *n* (%)**	
Stage 1	107 (42%)
Stage 2	110 (43%)
Stage 3	36 (14%)
**Time since diagnosis, years, mean (SD)**	3.0 (1.3)

Mean scores and standard deviations for sleep-related impairment (T-score) and self-reported cognitive outcomes (T-scores), as well as objective  cognitive domain composite z-scores and processing speed score are reported in Table 2. Descriptive severity categories based on PROMIS Sleep-related Impairment and PROMIS Cognitive Function T-scores cut points are presented in Table 3. Approximately 45.8% of participants reported sleep-related impairment within normal limits, while 21.3% reported mild impairment and 32.9% reported moderate-to-severe impairment. For cognitive function, 25.7% scored within normal limits, 38.7% reported mild cognitive difficulties, and 35.6% reported moderate-to-severe cognitive impairment.

**Table 2:** Mean PROMIS t-scores, objective cognitive domain composite z-scores, and processing speed score (*n* = 253)

Variable	Mean (SD)
PROMIS Sleep-related Impairment	55.78 (9.08)
PROMIS Cognitive Abilities	43.46 (7.28)
PROMIS Cognitive Function	41.40 (6.05)
Memory	0.00 (0.73)
Executive Function	0.00 (0.80)
Attention	−0.01 (0.51)
Processing Speed	77.09 (13.99)

**Table 3:** PROMIS T-score severity categories for sleep-related impairment and cognitive function (*n* = 253)

	**Within normal limits**	**Mild**	**Moderate**	**Severe**
PROMIS Sleep-related Impairment	116 (45.8%)	54 (21.3%)	73 (28.9%)	10 (4%)
PROMIS Cognitive Function	65 (25.7%)	98 (38.7%)	87 (34.4%)	3 (1.2%)

Higher PROMIS Sleep-Related Impairment T-scores indicate greater impairment, whereas lower PROMIS Cognitive Function T-scores indicate poorer perceived cognitive functioning

Results for the multiple linear regression models are reported in Table 4. Greater sleep-related impairment was significantly associated with worse executive function, although the magnitude of the association was small (*β* = −0.01, 95% CI: −0.022, −0.000, *p* = 0.04). For self-reported cognition, greater sleep-related impairment was significantly associated with worse cognitive abilities (*β* = −0.28, 95% CI: −0.375, −0.184, *p* < 0.001) and worse cognitive function (*β* = −0.25, 95% CI: −0.323, −0.167, *p* < 0.001). No significant associations were found between sleep-related impairment and objectively measured memory, attention, and processing Speed. Sensitivity analyses adjusting for age and education only had similar results (see Supplemental Table S1).

**Table 4:** Linear effects models of sleep-related impairment on cognition, controlling for covariates (*n* = 253)

Cognitive outcome	Estimate	Std error	95% CI	*p* value
PROMIS Cognitive Abilities	−0.28	0.05	(−0.375, −0.184)	< 0.001
PROMIS Cognitive Function	−0.25	0.04	(−0.323, −0.167)	< 0.001
Memory	0.00	0.01	(−0.008, 0.011)	0.78
Executive Functioning	−0.01	0.01	(−0.022, −0.000)	0.04*
Attention	−0.01	0.00	(−0.012, 0.002)	0.14
Processing Speed	−0.05	0.09	(−0.235, 0.134)	0.59

Covariates adjusted for: age, ethnicity (Hispanic vs. non-Hispanic), education (some college or less vs. college graduate or more), marital status (never married, divorced/separated/widowed, married/living with partner); cancer treatment (chemotherapy and hormone therapy, chemotherapy only , hormone therapy only), years since diagnosis, radiation (yes/no)

## Discussion

This study found that sleep-related impairment was associated with self-reported cognition and objectively measured executive function, but not all aspects of cognition. Our findings for self-reported cognitive function are consistent with recent literature, which suggests that poorer sleep is linked to self-reported cognitive function [14]. Notably, despite the variability of the self-report cognitive functioning measures used across studies, the consistent finding that poor sleep correlates with worse perceived cognitive functioning suggests that sleep disturbances may universally impact cancer survivors’ perceptions of cognitive functioning. Additionally, studies evaluating various types of sleep problems—including sleep quality, insomnia, and sleep disturbance—all find significant associations with self-report cognitive functioning [9, 17, 18, 23, 41]. This indicates that various sleep issues are related to how cancer survivors perceive their cognition, regardless of specific sleep domains or characteristics assessed. Although causality cannot be inferred from the present study, these findings support the importance of considering sleep-related daytime impairment within the broader context of survivorship symptom burden and cognitive functioning.

Participants in this sample experienced elevated sleep-related daytime impairment, with approximately one third of the sample reporting moderate-to-severe sleep-related impairment. These findings suggest that many survivors in this sample experienced meaningful daytime symptoms potentially affecting fatigue, alertness, and daily functioning. Elevated sleep impairment among survivors could stem from a myriad of factors, including a fear of recurrence or distress from physical, emotional, and economic challenges relating to cancer [42] that disproportionately impact this population. Clinically, sleep impairment among cancer survivors is associated with reduced quality of life, greater severity of pain, and physical function limitations [43–45]. Thus, screening for sleep-related impairment among cancer survivors may be an important clinical consideration during survivorship care. Perceived cognitive difficulties were also common, with more than a third of participants reporting moderate-to-severe perceived cognitive difficulties, and an additional 38.7% reporting mild cognitive concerns. Together, these findings highlight the burden of perceived cognitive symptoms among BCS following treatment. These self-reported cognitive deficits can adversely affect survivors’ everyday functioning, medication management, work productivity, and overall quality of life [46]. Therefore, identifying and assessing risk factors associated with cognitive difficulties, including sleep-related daytime impairment, may help support earlier recognition and management of CRCD among survivors.

Of the four objective cognitive outcomes assessed, sleep-related impairment was only significantly associated with one domain: executive function. Consistent with our findings, several studies have demonstrated significant associations between sleep problems (i.e., quality, disturbances, and insomnia) and objective executive functioning among cancer survivors [17, 21, 47], reinforcing the critical relationship between sleep, cognitive health, and daily functioning. Executive function encompasses three core capabilities: inhibitory control, working memory, and cognitive flexibility, which collectively support essential skills such as reasoning, problem-solving, and planning [47–50]. These abilities are critical for maintaining mental and physical health, managing daily responsibilities, and achieving overall quality of life [49]. Among breast cancer survivors, these cognitive skills are of particular importance as they face unique vulnerabilities regarding reduced executive functioning that may adversely affect their quality of life, self-efficacy, and ability to cope and regulate emotions [51]. The mechanisms by which sleep-related impairment is connected to executive function are complex and multifactorial [52]. Further research incorporating subjective objective sleep measures is needed to better understand the pathways linking sleep-related impairment and executive function among BCS.

Contrary to our hypotheses, sleep-related impairment was not significantly associated with objective memory, attention, or processing speed. These findings diverge from a substantial body of evidence linking sleep disturbances to objective memory deficits [14, 17, 53, 54] and attention impairments [54–56] among cancer survivors. Notably, our findings regarding processing speed align with previous studies that found no relationship between sleep-related difficulties and objective measures of processing speed in this population [10, 17, 54], despite deficits in processing speed being well documented among cancer survivors. Taken together, these results highlight the complexity of sleep-cognition relationships in cancer survivorship and underscore the need for future research using more diverse samples and sensitive measures for both sleep and cognitive function. 

### Study limitations

The results of our study should be interpreted within the context of several limitations. First, the majority of our sample is non-Hispanic White, college-educated individuals with high incomes, which may restrict the generalizability of our findings to more diverse populations. Second, the cross-sectional nature of the study prevents causal inference, limiting our ability to determine directionality of the relationship between sleep-related impairment and objective and perceived cognitive functioning. Third, while the PROMIS Sleep-Related Impairment scale is a valuable measure in understanding how sleep disruption affects cognitive functioning and daily activities, it does not directly assess sleep quality, patterns, or disturbances but rather self-reported perceptions of alertness, sleepiness, and tiredness during waking hours. Fourth, participants in the parent study were recruited to join a physical activity intervention and had to have low levels of activity to be eligible, which may have influenced the findings. Because objective baseline measures of moderate-to-vigorous physical activity were not available for the present analysis, we were unable to further evaluate the potential influence on these associations.

### Implications for cancer survivors

Despite its limitations, this study offers important implications for cancer survivors. Screening for perceived daytime impairments related to sleep and associated functional concerns may help identify survivors experiencing cognitive difficulties during survivorship. Given the pervasiveness of sleep-related symptoms and cognitive complications among cancer survivors, incorporating assessment of sleep-related impairment into survivorship care may support more comprehensive, patient-centered symptom management.

## Conclusion

In this sample of post-treatment breast cancer survivors, greater sleep-related impairment was associated with lower perceived cognitive abilities and functioning, as well as worse performance on objective measures of executive function. There were no significant relationships between sleep-related impairment and other objective measures of attention, memory, or processing speed. Findings suggest that sleep-related impairment may be more strongly associated with perceived cognitive experiences than with objective neuropsychological performance. The association with executive functioning indicates that sleep-related impairment is related to more complex cognitive domains, like reasoning and problem-solving, which may result in difficulties managing daily tasks. Further longitudinal research is warranted to understand the causal mechanisms by which sleep impacts cognition and how mood, fatigue, and quality of life may play a role in this relationship.

## Supplementary Information

Below is the link to the electronic supplementary material.

ESM 1 (15.6 KB DOCX)

## Data Availability

The raw data supporting the conclusions of this article will be made available by the authors, without undue reservation.
