# Supplementary material for: Relationship between sleep-related impairment and cognitive functioning among breast cancer survivors
Source: J Cancer Surviv. Author manuscript; Available in PMC 2026 Aug 3. (PMC13432458; doi:10.1007/s11764-026-02057-7)
Supplement: Supplemental Table [file NIHMS2194195-supplement-Supplemental_Table.docx]

| **Supplemental Table.** Sensitivity analyses examining associations between sleep-related impairment and cognitive outcomes, controlling for covariates (n=253) | | | | |
| --- | --- | --- | --- | --- |
| **Cognitive outcome** | **Estimate** | **Std Error** | ***95% CI*** | ***p* value** |
| PROMIS Cognitive Abilities | -0.28 | 0.05 | (-0.379, -0.194) | **<0.001** |
| PROMIS Cognitive Function | -0.25 | 0.04 | (-0.330, -0.178) | **<0.001** |
| Memory | 0.00 | 0.00 | (-0.010, 0.010) | 0.983 |
| Executive Functioning | -0.01 | 0.01 | (-0.023, -0.002) | **0.021*** |
| Attention | -0.01 | 0.00 | (-0.012, 0.002) | 0.128 |
| Processing Speed | -0.08 | 0.09 | (-0.260, 0.102) | 0.393 |
| ^*p < 0.05; Covariates adjusted for: Age and education (some college or less vs. college graduate or more)^ | | | | |
